# Bridging clinic and wildlife care with AI-powered pan-species computational pathology

**DOI:** 10.1038/s41467-023-37879-x

**Published:** 2023-04-26

**Authors:** Khalid AbdulJabbar, Simon P. Castillo, Katherine Hughes, Hannah Davidson, Amy M. Boddy, Lisa M. Abegglen, Lucia Minoli, Selina Iussich, Elizabeth P. Murchison, Trevor A. Graham, Simon Spiro, Carlo C. Maley, Luca Aresu, Chiara Palmieri, Yinyin Yuan

**Affiliations:** 1 Centre for Evolution and Cancer, The Institute of Cancer Research, London, UK; 2 Division of Molecular Pathology, The Institute of Cancer Research, London, UK; 3 Department of Veterinary Medicine, University of Cambridge, Madingley Road, Cambridge, UK; 4 Zoological Society of London, London, UK; 5 Centre for Genomics and Computational Biology, Barts Cancer Institute, Queen Mary University of London, Charterhouse Sq, London, UK; 6 Department of Anthropology, University of California Santa Barbara, Santa Barbara, CA USA; 7 Department of Pediatrics and Huntsman Cancer Institute, University of Utah, Salt Lake City, UT USA; 8 PEEL Therapeutics, Inc., Salt Lake City, UT USA; 9 Department of Veterinary Sciences, University of Turin, 10095 Grugliasco, Italy; 10 Arizona Cancer Evolution Center, Biodesign Institute and School of Life Sciences, Arizona State University, Tempe, AZ USA; 11 School of Veterinary Science, The University of Queensland, 4343 Gatton, QLD Australia; 12 Present Address: Department of Translational Molecular Pathology, The University of Texas MD Anderson Cancer Center, Houston, TX USA

**Keywords:** Cancer imaging, Machine learning, Zoology, Tumour immunology, Pathology

## Abstract

Cancers occur across species. Understanding what is consistent and varies across species can provide new insights into cancer initiation and evolution, with significant implications for animal welfare and wildlife conservation. We build a pan-species cancer digital pathology atlas (panspecies.ai) and conduct a pan-species study of computational comparative pathology using a supervised convolutional neural network algorithm trained on human samples. The artificial intelligence algorithm achieves high accuracy in measuring immune response through single-cell classification for two transmissible cancers (canine transmissible venereal tumour, 0.94; Tasmanian devil facial tumour disease, 0.88). In 18 other vertebrate species (mammalia = 11, reptilia = 4, aves = 2, and amphibia = 1), accuracy (range 0.57–0.94) is influenced by cell morphological similarity preserved across different taxonomic groups, tumour sites, and variations in the immune compartment. Furthermore, a spatial immune score based on artificial intelligence and spatial statistics is associated with prognosis in canine melanoma and prostate tumours. A metric, named morphospace overlap, is developed to guide veterinary pathologists towards rational deployment of this technology on new samples. This study provides the foundation and guidelines for transferring artificial intelligence technologies to veterinary pathology based on understanding of morphological conservation, which could vastly accelerate developments in veterinary medicine and comparative oncology.

## Abstract (web summary)

Artificial Intelligence (AI) has the potential of assisting the study and diagnosis of veterinary cancers. Here, the authors build a cancer digital pathology atlas encompassing multiple animal species and demonstrate an AI approach for comparative pathology, which yields insights about immune response and morphological similarities.

## Introduction

Cancers occur with phenotypically similar forms across the tree of life^1–4^. Understanding the conserved and diverged aspects of cancer across species can help answer questions about the origin and fundamental processes of its evolution. Immediate and practical advances from pan-species studies provide new tools and valuable insights into tumorigenesis and cancer resistance^5–8^, leading to improved cancer care for humans and non-human animals^9^. Specifically, transmissible cancers presented in dogs and Tasmanian devils^10,11^ are among the few known naturally occurring clonally transmissible cancers^12^. How transmissible cancers escape immune surveillance remains unclear and is of central importance to understanding their biology and cell-to-cell interactions.

Despite significant resources in companion animal care, clinical treatment options are limited for a few aggressive cancers in dogs^13,14^ that represent one of the best models of human cancer^15^. Beyond domesticated species, various studies have identified valuable models in wildlife^16^. For instance, the naturally-emerging urogenital carcinoma in California sea lions^17^ and papillomavirus triggering brain tumours in raccoons^16^ are remarkable examples of pathogen-driven neoplasms. Animals managed in zoological institutes also exhibit occurrence of neoplastic growth according to several international studies, including a 10-year survey in the Taipei zoo, Taiwan^18^, a study of cancer development in vertebrates in French zoological parks^19^, a 42-year of mammals necropsy data compilation from the San Diego Zoo, United States^20^, and a report on renal lesions followed by neoplastic and inflammatory responses in captive wild felids in Germany^21^. Studies of these animals can provide unique insights into the biology and evolution of cancer across the tree of life towards improving animal welfare by early detection and helping conserve endangered species^22,23^.

Challenges for establishing a unified comparative oncology agenda include sample collection, data management, analysis, and integration^24–29^. These can be tackled by incorporating artificial intelligence (AI) algorithms, which can empower veterinary pathology and help dissect the complexity of cancer across species and scales, from genes to epidemiology. Computational pathology powered by AI has revolutionised the study of human cancers and helped improve our understanding of the immune microenvironment (e.g., ^30^). In contrast to human cancer management, we lack systematic and standardised AI protocols and digital archiving and analysis of samples to study animal cancers. Therefore, veterinary research has not fully adopted digital pathology^26^ although efforts are being made to move forward internationally adopted guidelines for tumour pathology^28^.

Hence, we propose AI has the power to fuel pan-species tumour histology and efficiently manage data-related bottlenecks. Thus far, computational pathology in the study of non-human cancers, and non-human pathology in general, is very limited^25,26^. Convolutional neural networks have been applied to detect mitotic activity from histological slides of canine cancers^14,31^. In sheep, deep learning has been employed to delineate the growth phases of mammary development^32^. Other machine learning techniques have been used to classify a common gastrointestinal disease in cats^33^. Along with computational pathology, incorporating AI into the veterinary practice of imaging techniques such as CT scans, magnetic resonance imaging, and positron emission tomography^34^ encourages the development of integrative clinical care. Such an integrative approach promises to direct precision medicine in veterinary oncology by tailoring strategies for individual patients. It includes classifying patients who differ in their treatment response and/or prognostic outcomes.

**Fig. 1:**
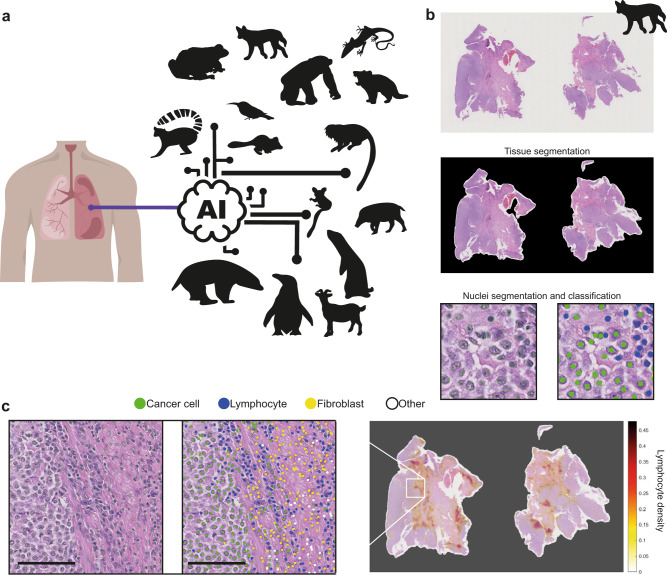
Pan-species computational pathology. **a** Transfer learning of cell identification from human lung to pan-species tumour pathology. **b, c** Overview of the H&E single-cell analysis pipeline as illustrated using a dog’s (CANFAM) transmissible venereal tumour sample. **b** This AI pipeline^35^ first segments the viable tissue area, then detects and classifies all cells into cancer, stromal, lymphocyte and others. For more details, see Methods. **c** The pipeline is implemented to spatially profile the immune microenvironment at the whole-slide level (right, immune cell density, cells/pixels^2^), after single-cell segmentation (left) and cell classification (middle). Scale bar, 50 µm. Cell colours are denoted as four classes, green: cancer (malignant epithelial) cells; blue: lymphocytes (including plasma cells); yellow: noninflammatory stromal cells (fibroblasts and endothelial cells); white: ‘other’ cell class that included nonidentifiable cells, less abundant cells such as macrophages and chondrocytes and ‘normal’ pneumocytes.

In this work, we explore and exploit the conservatism of cell morphology in tumours across species by applying an AI tool trained in human lung cancer^30^ (Fig. 1). We evaluate: (1) the generalisability and accuracy of this AI model in mapping tumour cell distribution and lymphocytic infiltration in histological tissues from transmissible cancers and 18 other species; and (2) the prognostic value of immune infiltration, particularly through accounting for cell spatial co-occurrence between lymphocytes and cancer cells^35^, in canine melanoma and prostate carcinoma tissue sections^36–39^. In this work, we apply computational pathology algorithms to transmissible cancers and pan-species pathology beyond mammals, thereby decoding the composition of cells in tumours across species. Our approach aims to pave the way for pan-species comparative pathology and contribute to a basic understanding of the emergence and prevalence of cancer in nature.

## Results

### Collection of veterinary histology samples

We present a publicly available pan-species digital pathology atlas that includes 120 hematoxylin and eosin (H&E) digital slide images and 41,567 pathological single-cell annotations, specifically curated for veterinary computational pathology (panspecies.ai).

First, for the evaluation of AI performance, a set of 99 H&E samples from 18 species was selected and digitalised from the Zoological Society of London’s (ZSL) pathological archive (classes, Mammalia = 11 species, Reptilia = 4, Aves = 2, and Amphibia = 1). In addition, four tumour samples from Tasmanian devils with facial tumour disease 1 and 2 (DFT1 and DFT2) and seven with canine transmissible venereal tumour (CTVT) were collected and digitalised from the Transmissible Cancers Group, University of Cambridge. Of all these, 58 images of 20 species passed quality control for image analysis. The pathologists selected one representative slide for each species spanning across Mammalia, Reptilia, Aves, and Amphibia to produce a combined ‘cohort 0’ of 20 H&E sections from 20 different species categorised per neoplastic type as round-cell (*n* = 4), epithelial (*n* = 9), mesenchymal (*n* = 4), neuroendocrine (*n* = 2) and sex-cord stromal (*n* = 1) tumours. Secondly, to test the prognostic value of immune infiltration measured by AI and spatial statistics, we utilised 100 H&E images from a canine melanoma cohort (*n* = 88, provided by the University of Turin) and a canine prostate carcinoma cohort (*n* = 12, provided by the University of Queensland) with clinical outcome data.

### Transferring AI technologies to non-human species

The AI human-lung model, a deep-learning pipeline tailored for human lung cancer (predominantly lung adenocarcinoma, including lung squamous cell carcinoma^30^, Fig. 1a) was applied without modification to all pan-species 120 H&E samples (cohort 0 *n* = 20, melanoma cohort *n* = 88, and prostate cohort *n* = 12). Briefly, this pipeline identifies the precise location of individual cells in each H&E and classifies them based on nuclear morphological and staining features in one of four cell types: cancer cells, lymphocytes, stromal cells (fibroblasts and endothelial cells) and ‘other’ cells (macrophages, pneumocytes and nonidentifiable cells) (Fig. 1b, c). To evaluate the accuracy of cell classification, we compared its predictions against veterinary pathologists’ annotations.

**Fig. 2:**
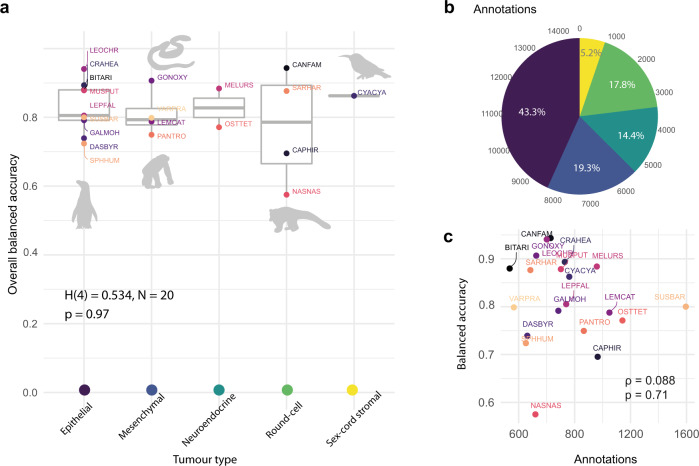
AI single-cell prediction comparison across tumour types. Balanced accuracy is computed as the average of sensitivity and specificity, ‘overall’ refers to the average of cancer, stromal and lymphocyte cells. **a** A Kruskal–Wallis test, *n* = 20, was used to compare the overall balanced accuracy between tumour types. Thick horizontal lines indicate the median value, outliers are indicated by the extreme points, the first and third quantiles are represented by the box edges, and vertical lines indicate minimum and maximum values. **b** Distribution of the number of annotations by tumour type (colours correspond to tumour types in **a**). **c** Relationship between the number of annotations and the overall balanced accuracy for each species (*n* = 20) using a Spearman’s correlation. Species in (**a**) and (**c**) are labelled with their codes; for more information, see Table 1.

We first evaluated the accuracy of the human-lung model across all 20 species from cohort 0, using 14,570 cancer, lymphocyte, and stromal single-cell annotations provided by two board-certified specialist veterinary pathologists (C.P. and K.H.). For each slide, we computed the algorithm’s balanced single-cell classification accuracy (BCAcc, Table 1), as well as F1 score, precision, sensitivity, specificity (Supplementary Figs. 1 and 2), corresponding confusion matrix (Supplementary Fig. 3), and the AUROC values per cell class (Supplementary Table 1). The algorithm’s average BCAcc across cell classes showed a diverse variation between and within tumour groups (Fig. 2a, Supplementary Figs. 1 and 2). Tumour types had similar overall BCAcc values (Kruskal–Wallis test, H(4) = 0.534, *n* = 20, *p* = 0.97, *η*^2^ = −0.231, 95% CI = −0.21 to 0.49). Despite a variable number of annotations per tumour type (Fig. 2b), BCAcc was not associated with the number of annotations (Spearman’s *ρ* = 0.088, p = 0.71) (Fig. 2c).

**Table 1:** Summary of overall balanced classification accuracy (BCAcc) by species. Balanced accuracy is computed as the average of sensitivity and specificity for cancer, stromal and lymphocyte cells

Code	Common name	Scientific name	Diagnosis	Neoplasia site	Tumour type	Annotations	BCAcc
BITARI	Puff adder	*Bitis arietans*	Carcinoma	Pancreas	Epithelial	336	0.88
CANFAM	Dog	*Canis lupus familiaris*	Canine transmissible venereal tumour	Intra vaginal	Round-cell	629	0.94
CAPHIR	West African pygmy goat	*Capra hircus*	Lymphoma	Forestomach	Round-cell	965	0.70
CRAHEA	Panay cloudrunner	*Crateromys heaneyi*	Hepatocellular carcinoma	Liver	Epithelial	730	0.89
CYACYA	Red-legged honeycreeper	*Cyanerpes cyaneus*	Sertoli cell tumour	Testis	Sex-cord stromal	762	0.86
DASBYR	Kowari	*Dasyuroides byrnei*	Squamous cell carcinoma	Mouth	Epithelial	462	0.74
GALMOH	Mohol bushbaby	*Galago moholi*	Squamous cell carcinoma	Skin	Epithelial	684	0.79
GONOXY	Red-tailed green ratsnake	*Gonyosoma oxycephala*	Metastatic anaplastic sarcoma	Multiple	Mesenchymal	526	0.91
LEMCAT	Ring-tailed lemur	*Lemur catta*	Haemangiosarcoma	Kidney	Mesenchymal	1049	0.79
LEOCHR	Golden-headed lion tamarin	*Leontopithecus chrysomelas*	Adenoma	Pituitary	Epithelial	601	0.94
LEPFAL	Mountain chicken	*Leptodactylus fallax*	Adenocarcinoma	Celomic cavity	Epithelial	740	0.81
MELURS	Sri Lankan sloth bear	*Melursus ursinus inornatus*	Pheochromocytoma	Adrenal	Neuroendocrine	959	0.88
MUSPUT	Domestic ferret	*Mustela putorius furo*	Sebaceous epithelioma	Skin	Epithelial	702	0.88
NASNAS	South American coati	*Nasua nasua*	Lymphoma	Multiple	Round-cell	520	0.57
OSTTET	African dwarf crocodile	*Osteolaemus tetraspis tetraspis*	Lipoma	Liver	Neuroendocrine	1142	0.77
PANTRO	Chimpanzee	*Pan troglodytes*	Spindle cell tumour	Palate	Mesenchymal	866	0.75
SARHAR	Tasmanian devil	*Sarcophilus harrisii*	Devil facial tumour 1 (DFT1)	Hard palate near left side	Round-cell	484	0.88
SPHHUM	Humboldt penguin	*Spheniscus humboldti*	Renal cell adenoma	Kidney	Epithelial	452	0.72
SUSBAR	Bearded Pig	*Sus barbatus*	Adenocarcinoma	Uterus	Epithelial	1595	0.80
VARPRA	Emerald Tree monitor	*Varanus prasinus*	Spindle cell sarcoma	Multiple	Mesenchymal	366	0.80

To determine the accuracy of this model on the clinical datasets, a board-certified veterinary pathologist (C.P.) provided 26,997 single-cell annotations (13,177 cancer cells, 10,312 lymphocytes, and 3508 stromal cells) on the canine prostate carcinoma cohort. An average BCAcc of 0.9 was observed across the three cell classes (cancer cells = 0.88, lymphocytes = 0.95, and stromal cells = 0.87; Supplementary Table 2). In particular, we observed a higher sensitivity and specificity for classifying lymphocytes compared to cancer and stromal cells (Supplementary Table 2).

To evaluate if an alternative method trained from animal annotations could achieve a better transferability across species compared with the human-lung model, we utilised the 26,997 single-cell annotations from the canine prostate carcinoma cohort to train a new deep-learning model (referred to as the canine carcinoma prostate model) using the same architecture as the human-lung model and tested it across all 20 species from the cohort 0. The overall BCAcc averaged across samples was 0.62 (range: 0.496–0.797), compared to an average of 0.81 (range: 0.57–0.0.94) from the human-lung model. In particular, when applied to a target sample of the same species from cohort 0 (dog’s CTVT), BCAcc was 0.53, compared to 0.94 achieved using the human-lung model (Supplementary Table 3). When applied to a target sample of the same species from cohort 0 (CTVT), BCAcc was 0.53, compared to 0.94 achieved using the human-lung model (Supplementary Table 3). These data suggest that an AI model trained on non-human species is not necessarily superior to AI models trained on human samples when it comes to veterinary applications.

### Consistent accuracy in classifying cancer cells and lymphocytes

**Fig. 3:**
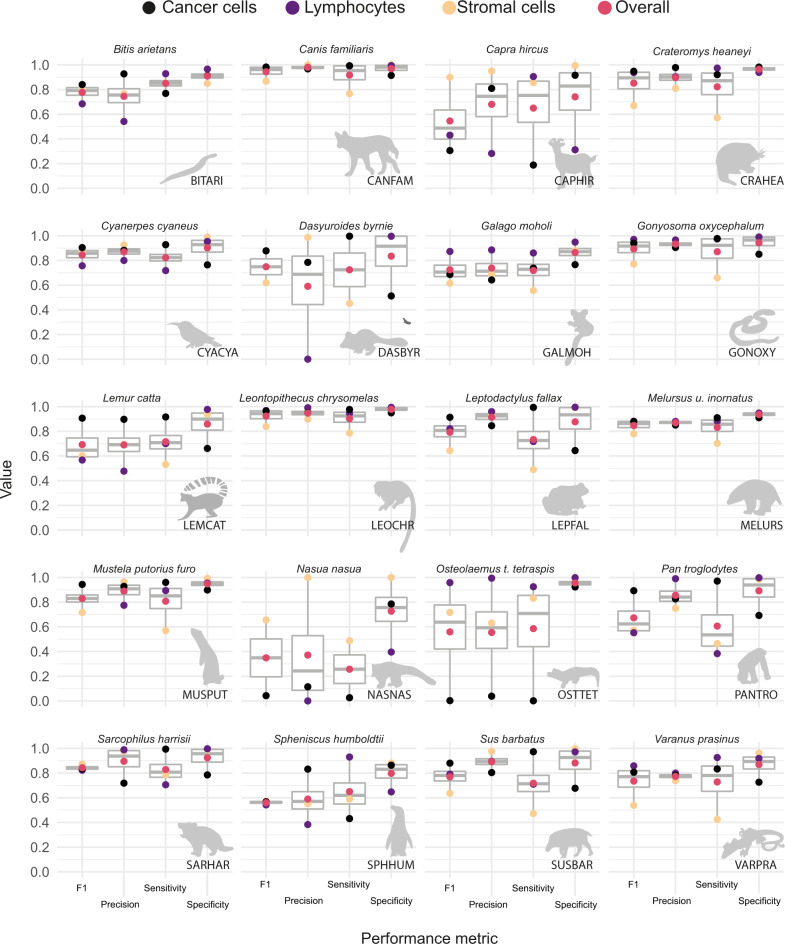
AI model prediction performance metrics for 20 species. For each species, four metrics were evaluated, including F1, precision, sensitivity, and specificity (as labelled on the x-axis) for the prediction accuracy of cancer, lymphocytes, and stromal cells, as well as their average shown as ‘overall’ (as denoted with colours on the top x-axis). For species codes, see Table 1. For the number of cells annotated per cell class for each species, see Supplementary Table 1. Thick horizontal lines indicate the median value, outliers are indicated by the extreme points, the first and third quantiles are represented by the box edges and vertical lines indicate minimum and maximum values.

Overall, the model’s best performance was for mammalian tumours within our limited dataset in cohort 0 (Fig. 3, Supplementary Figs. 2 and 3). In particular, the AI algorithm achieved high accuracy for the two transmissible cancers (CTVT, 0.94; DFT1, 0.88). Furthermore, the CVTV (in *Canis lupus familiaris*) exhibited the best accuracy across all 20 species (overall precision = 0.98, F1 and BCAcc = 0.94, Fig. 3). In the 18 other vertebrate species from the ZSL cohort, overall BCAcc varied between 0.57 and 0.94. The performance of cancer cell and lymphocyte classification, measured as BCAcc, did not vary between tumour types (Kruskal–Wallis test, cancer cell: *H*(4) = 0.72, *n* = 20, *p* = 0.95, *η*^2^ = −0.22, 95% CI = −0.22 to 0.46; lymphocyte: *H*(4) = 0.534, *n* = 18, *p* = 0.74, *η*^2^ = −0.157, 95% CI = −0.19 to 0.61). However, the BCAcc of stromal cells differed between tumour types (Kruskal–Wallis test, cancer cell: *H*(4) = 9.52, *n* = 20, *p* = 0.048, *η*^2^ = 0.37, 95% CI = 0.08–0.73), with pairwise comparisons significant for differences between epithelial vs round-cell tumours (z-test, z(15) = −2.34, *p* = 0.018, estimate = −0.088, SE = 0.038, 95% CI = −0.17 to −0.008) and mesenchymal vs round-cell tumours types (z-test, z(15) = −2.6, *p* = 0.02, estimate = −0.116, SE = 0.045, 95% CI = −0.21 to −0.021). All other comparisons include 0 for the confidence interval of their differences. Surprisingly, in both cases where we reported significant differences, the balanced accuracy of stromal cells in round-cell tumour types was higher than in mesenchymal or epithelial tumour types. In the cohort 0, the round-cell tumour types were identified in the dog (*Canis l. familiaris*), the Tasmanian devil (*Sarcophillus harrisii*), the pygmy goat (*Capra hircus*), and the ring-tailed coati (*Nasua nasua*). These results show consistent accuracy in classifying cancer cells and lymphocytes across species, while the variability of AI performance is largely driven by stromal cells.

### Explaining the model’s accuracy using morphological overlap across species

**Fig. 4:**
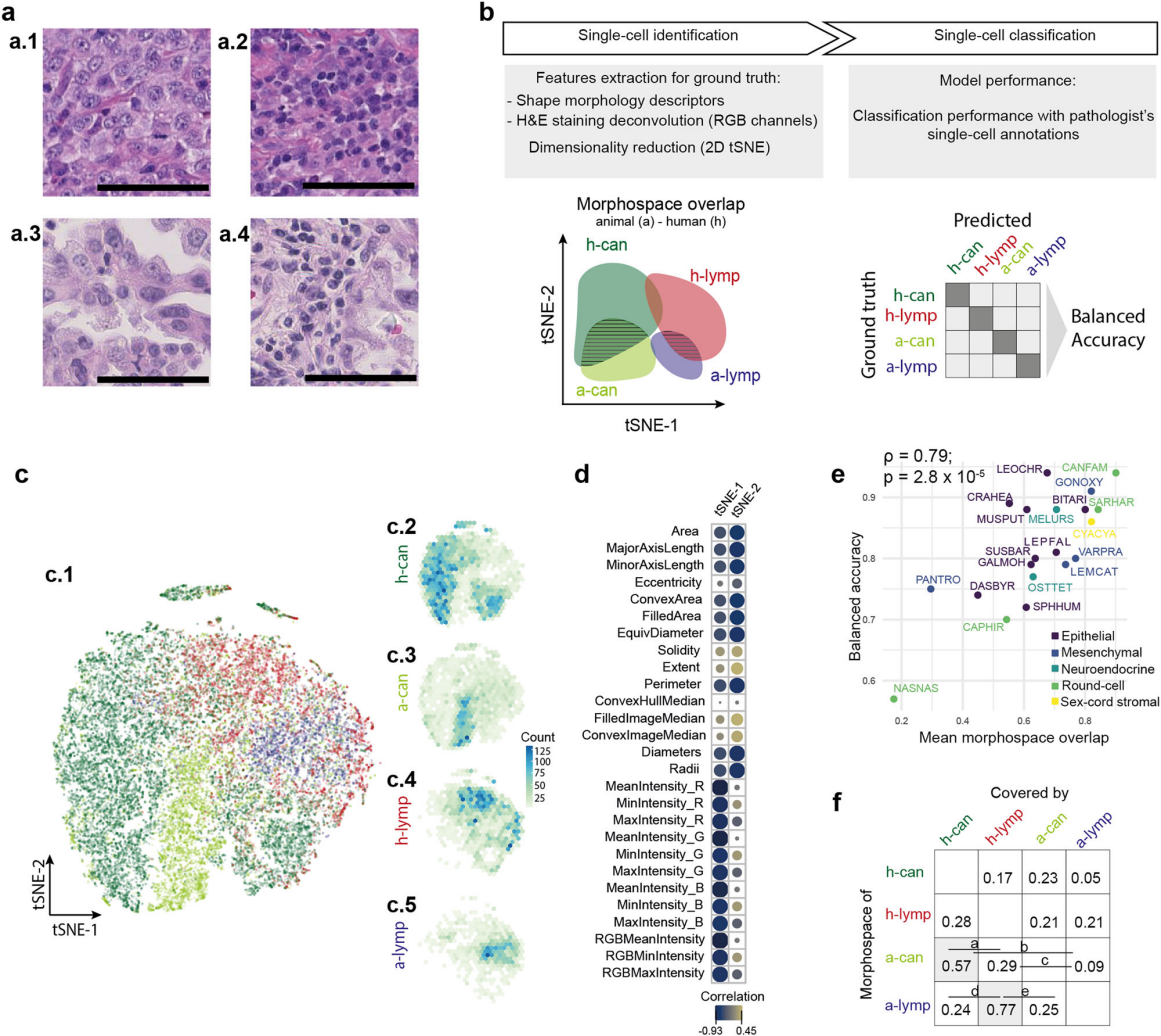
Overlap across the morphological space. **a** Canine (**a.1**, **a.2**) and human cells (**a.3, a.4**) have visible morphological similarities even for non-specific stainings such as hematoxylin and eosin across cancer cells (**a.1**, **a.3**) and immune cells (**a.2**, **a.4**). Scale bar = 50 µm. **b** Explanation of the morphospace overlap score based on feature extraction of single cells annotated by pathologists, human (h-) and animal (a-) cancer cells (can) and lymphocytes (lymp). **c** Dimensionality reduction enables the evaluation of morphospace overlap across human (**c.2**, **c.3**) and non-human cells (**c.4**, **c.5**). **d** The t-SNE dimensions show  explainable cell features associated with staining and morphology (see Supplementary Table 4). **e** Model accuracy correlates with the mean morphospace overlap for each sample in cohort 0 (Spearman’s correlation, *n* = 20). **f** Mean values of morphospace overlap between human and animal cells across all the 20 samples of the cohort 0. Animal lymphocytes (a-lymp) and cancer cells (a-can) have the highest morphospace overlap with human lymphocytes (h-lymp) and cancer cells (h-can), respectively (shadowed cells in the matrix). Letters indicate only statistically significant comparisons, row-wise, after multiple comparison with Benjamini & Hochberg correction (*p* values: *a* = 0.0004, *b* = 5.4 × 10^−9^, *c* = 0.0006, *d* = 6.4 × 10^−8^, *e* = 3 × 10^−6^).

To explore the morphological similarity between human and non-human cells and to explain the transferability of the human-lung model, we visualised and quantified the morphological space of ~32,000 cells annotated by expert pathologists (Fig. 4a, b). A t-distributed stochastic neighbour embedding (t-SNE) technique was used to reduce 27 single-cell features extracted by the AI human-lung model (Supplementary Table 4) to two dimensions, allowing us to develop a metric, named morphospace overlap, across cell classes and tumour types (Fig. 4b–d; Supplementary Table 6). Morphospace overlap measures the fraction of the morphospace of a cell class intersecting with a second cell class. We found that the human-lung model’s balanced accuracy positively correlated with mean morphospace overlap (Pearson’s *ρ* = 0.79, *p* = 2.8 × 10^−5^; Fig. 4e), suggesting a better model performance on species with higher cell morphological similarity with human cells from the training dataset. Interestingly, species/tumour-specific analyses revealed further insights into the model’s performance (Supplementary Table 5). Non-human cell classes have higher morphospace overlap with their human counterparts than other cells classes of the same species, suggesting that they are morphologically more similar to their human counterparts than other cell classes of the same species (Fig. 4f). Thus, morphospace overlap as a metric may be a useful tool as part of model selection and explanation for pathologists to determine the usability of our (or any other) AI tool.

### An AI-based immune score is prognostic in canine cancers

AI-based immune scores have been demonstrated to be prognostic in several human cancers^30,40–42^. However, their clinical relevance in veterinary medicine has not been tested so far. To evaluate the clinical utility of immune scores in non-human animals, we analysed the canine melanoma and prostate cohorts. Two immune scores were tested, including a quantitative measure of immune cell relative abundance (percentage of lymphocytes) and, since lymphocyte surveillance requires cell-cell interactions for effective immune synapses^39^, we evaluated an immune score based on spatial statistics that consider the location of lymphocytes and their proximity to cancer cells, the tumour-immune colocalisation score^35^ (for details see Methods).

**Fig. 5:**
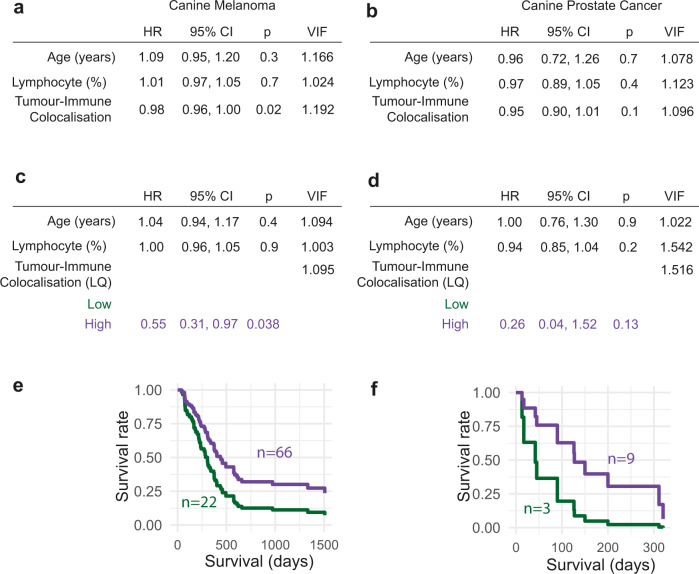
Prognostic value of spatial immune infiltration. **a**, **b** Multivariate Cox regression analyses testing the prognostic value of the colocalisation between cancer cells and lymphocytes as a continuous variable for overall survival in melanoma, *n* = 88 (**a**) and prostate canine cancers, *n* = 12 (**b**). Other variables included are age and AI-based lymphocyte percentage (see Methods). The same multivariate model but incorporating the colocalisation between cancer cells and lymphocytes as a dichotomised variable using the upper three quartiles as high group, lower quartile as low group, determined individually for melanoma (**c**), and prostate (**d**) canine tumours. LQ, lower quartile, HR, hazard ratio, CI, confidence interval, VIF, variance inflation factor. Kaplan–Meier curves illustrating the difference in overall survival according to the colocalisation between cancer cells and lymphocytes, dichotomised into high (upper three quartiles) and low (lower quartile) groups, in melanoma (**e**), and prostate (**f**) canine tumours. Higher tumour-immune colocalisation is associated with a better prognosis in melanoma patients. For prostate cancer, the same trend is observed (see Methods), although non-significant.

The tumour-immune colocalisation score, but not the immune abundance score, was found to be associated with survival in the canine melanoma cohort (*n* = 88, Fig. 5a, c). In a multivariate analysis controlling for age and lymphocyte relative abundance, a high tumour-immune colocalisation score, suggesting that lymphocytes in the tumour colocalise well with cancer cells, was associated with favourable overall survival (HR = 0.98, *p* = 0.02, *n* = 88; Fig. 5a). The same pattern was observed after dichotomising colocalisation (Fig. 5c, e, cut-off selected using lower quartile, high colocalisation HR = 0.55, *p* = 0.038 relative to low colocalisation). A similar trend was observed in the canine prostatic carcinoma dataset where a high tumour-immune colocalisation score was associated with good survival (*n* = 12, continuous colocalisation: HR = 0.95, *p* = 0.1; dichotomised colocalisation: HR = 0.26, *p* = 0.13, Fig. 5b, d, f). Despite the limited cohort sizes, these results highlight the potential importance of automated quantification and spatial analysis of immune infiltration for risk stratification in both canine melanoma and prostatic carcinoma.

## Discussion

Comparative oncology pursues the understanding of cancer as a shared phenomenon among species which can generate new insights for cancer biology and human cancers^9,43^. Here, we have explored the potential and limitations of AI through automated pathological image analysis to study cancer morphology and immune response across species. Previous studies have often been limited to a single species, with applications mainly focused on canine and mouse models (e.g.^8,14,44^). This work expands the study of computational pathology, including tumours from vertebrates beyond mammals, such as aves, reptiles and one amphibian. It also demonstrates the prognostic value of an AI-based spatial immune score for veterinary medicine.

**Fig. 6:**
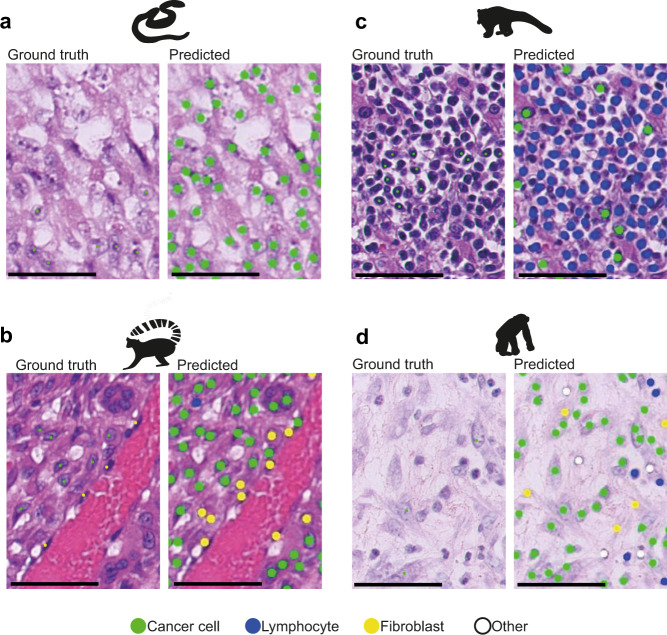
Strengths and pitfalls of current methods. Each H&E example is shown as a raw image with expert pathology annotations on some cells (Ground truth, left) and the AI cell identification (Predicted, right). Cell colours are denoted as four cell classes, green: cancer (malignant epithelial) cells; blue: lymphocytes (including plasma cells); yellow: noninflammatory stromal cells (fibroblasts and endothelial cells); white: ‘other’ cell class that included nonidentifiable cells, less abundant cells such as macrophages and chondrocytes and ‘normal’ pneumocytes. Scale bar = 50 µm. **a** Correct identification of cancer cells from a mesenchymal tumour (metastatic anaplastic sarcoma) in a snake (GONOXY). **b** A malignant spindle cell tumour from a ring-tailed lemur (LEMCAT) with a haemangiosarcoma disease, as shown, the neoplastic endothelial cells have large and rounded nucleus, which may appear morphologically similar to epithelial cancer cells, as opposed to the AI model’s own normal -stromal- endothelial cells. However, the model successfully distinguished the majority of neoplastic from stromal cells. Further complexity is in the occurrence of epithelioid haemangiosarcoma, where the cells of origin are endothelial cells but they became epithelial-like. **c** A challenging South American coati (NASNAS) case was diagnosed with a round-cell tumour (lymphosarcoma) where the cancer cell morphology is difficult to be recognised by an algorithm trained with epithelial cells from human lung cancer. **d** In the case of a chimpanzee (PANTRO) with a spindle cell sarcoma, the neoplastic fibroblasts are harder to differentiate from reactive fibroblast.

Although the algorithm was trained on human samples, it was able to distinguish three major cell types with high accuracy in many species (19/20 species in the pan-species cohort reached an accuracy ≥70% and 12/20 species ≥80%; Fig. 6). In line with these observations, the human lung model’s transferability was overall superior across the 20 species, when compared to a different model trained from canine prostate carcinoma tissue. A number of factors may underpin this observation, including the large-scale study design for training and testing AI, long development cycles and computational resources, stringent quality control and management protocols for human samples, making it difficult to match within the currently available veterinary medicine. Our results highlighted the importance of quantitative evaluation of AI applications in veterinary pathology. Although in many species, our AI tool did not achieve sufficiently high accuracy, we developed a quantitative metric, namely the morphospace overlap index, to guide future efforts of transfer learning. Transfer learning from AI trained on other species, controlled by carefully chosen, standardised metrics such as the morphospace overlap index, can be a useful tool for veterinary medicine. Broadly, our comparative analysis revealed that morphological conservation across certain species dictates that cells can be detected and correctly classified by a human specimen-trained AI, fostering our endeavour to develop pan-species computational pathology.

A potential impact of this study is to empower precision medicine for managing and treating animal cancers. Accurate diagnosis and timely treatment could be critical in preserving endangered and threatened species representing important breeding populations^45^. We demonstrated how the AI tool can be used to study lymphocytic infiltration in canine transmissible venereal tumours, melanoma and prostate tumours and in Tasmanian devil facial tumours with high accuracy and spatial resolution of single-cell locations. As a transmissible disease, the immune response at the organismal level may offer new alternatives to understanding the spread of the disease at a population scale from an epidemiological perspective^46,47^. Cancer cells from these tumours can colonise a new host by crossing the barriers of histocompatibility associated with the immune system and expressing immunosuppressive cytokines^48,49^. The quantification and spatial detection of both cancer and immune cells can help study immune evasion and treatment in transmissible cancers, building on progress in understanding T cells immune infiltration in Tasmanian devils^50^ and immune regulation in CTVT tumour regression^51^.

Furthermore, we showed that scoring of immune infiltration may have clinical relevance in canine melanoma and prostate tumours, potentially driven by immune synapses between effector T cells and cancer cells^39^. Previously, immune-related features such as lymphocyte subsets and PD-L1 scores have been associated with survival in canine melanoma, prostate cancer, and lymphoma^36,38,52,53^. Here, we demonstrated the clinical utility of immune scoring based on routine H&E samples, representing a cost-effective and streamlined solution for risk stratification, much like initiatives in human cancers^54,55^. This is also particularly important for animal cancer management^56^, because of the limitation in resource and cost constraint, for example, owners of companion animals may not want to consider tests with high costs. In addition, our finding resonates with recent discoveries in human cancers that the spatial context of immune cells is crucial to cancer development and response to therapy^30,40,54,57,58^. AI facilitates the integration of histology spatial context and the discovery of quantitative scores relevant to the cancer of focus, as demonstrated in our study. The immune spatial score that accounts for immune cell colocalisation to cancer cells was prognostic in a canine melanoma dataset and showed a similar trend for a small prostate cancer dataset. In contrast, a measure of lymphocyte percentage in the tumour was not. Thus, a detailed study of the tumour microenvironment facilitated by AI can guide new discoveries to understand the mechanisms behind survival, sensitivity, and resistance to standard treatments such as chemotherapy^59^. By enabling precision medicine, we can advance towards a more personalised and integrative approach to veterinary care^60^.

Medical treatment for animals has dramatically improved in veterinary clinics, zoological institutions and even wildlife veterinarians^61^, leading to better options for diagnosing and treating cancer in animal patients^24^. Despite these significant advances in veterinary oncology^62^, there are crucial constraints and limited availability of veterinary specialists^63^, and consequently, digital tools are not widely used^25,26^. Thus, computational pathology for different species and tumour types will bring tremendous advances for clinical veterinary care and comparative oncology research^25,64^. Many of the advantages are similar to those for human pathology, with the greatest benefits being accessibility to veterinary pathologists, time saved and increased diagnostic accuracy, however, significant challenges remain. For instance, the low rate of samples passing quality control highlights a marked difference in sample management between veterinary and human cancer care. Therefore, the resources provided in this research (see panspecies.ai) represent a significant step towards an efficient and cost-effective transfer of AI technologies to veterinary medicine.

We further observed that the transferability performance of the human-lung model was limited in some cases. Morphologically complex cancers that exhibit drastically different morphological features compared to epithelial histotypes or cancers with a high degree of similarity to normal cells represent significant hurdles for transfer learning. The immune compartment, which is highly variable among mammals, birds and reptiles^65^, imposes difficulties that seem complicated to pass with a generic algorithm. Moreover, this is amplified when evaluating cancer affecting the haematopoietic tissue, such as lymphoma in the coati (*N. nasua*, Fig. 6c) and pygmy goat (*C. hircus*). Similarly, for the chimpanzee (*Pan troglodytes*) with a spindle cell sarcoma, the neoplastic fibroblasts were hard to differentiate from reactive fibroblasts (Fig. 6d). This is a challenge both for the automated analysis and manual identification by pathologists. In those cases, it may be appropriate to take alternative strategies such as re-train a model, testing available cancer-specific models or developing a new model incorporating other tissue characteristics. To address the issue of model suitability, explain the model’s accuracy, and avoid unnecessary single-cell classification, we developed a metric, named morphospace overlap, to guide pathologists who wish to apply the AI tools to their samples based on morphological similarity obtained after single-cell detection. Based on our data, the transferability of existing AI technologies developed for humans to the veterinary domain may be significantly higher than previously thought.

Limitations of our study include the limited availability of samples and annotations. It will be important to validate our findings on extended pan-species cohorts and advance our understanding of intratumor heterogeneity across different species and derive more controlled interspecies comparisons. For example, future attempts can shed more light on immune compositions in the microenvironment using detailed multiplexing profiles. Nevertheless, this work represents a step forward towards a future where machine learning is incorporated in diagnostic investigations of natural and emerging diseases in non-human animals, enhancing accuracy and sensitivity and complementing veterinary pathologists’ capability in the decision-making process. Computational pathology can bring valuable opportunities for automated diagnosis, tumour grading, scoring, and precision medicine for animal cancers.

Finally, comparative oncology also brings tremendous benefits to human cancer research^5,9,43,66,67^. The understanding of cancer in model animals is crucial for the selection of the right animal models^68^ for human diseases and the pre-clinical development of drugs^69^. This knowledge is also being transferred into new therapeutic approaches for animals themselves to guide the rational application of immunotherapy^70^. Our knowledge of cancer in wild animals is limited, and computational pathology can greatly expand research opportunities that compare cancer in the wild to managed populations, as well as comparisons with human cancer. Cross-species cancer comparisons may help address fundamental questions in cancer biology and evolution too. This work revealed highly conserved morphology features across many species, particularly in epithelial and round-cell tumours, highlighting potential evolvability constraints for certain tumour types. The mismatch between species’ evolutionary history and the conserved cellular morphological diversity raises new questions on the origin of cell morphological patterns; is morphological conservation fixed early in metazoan evolutionary history? Or is it the result of stabilising selection imposed by the extracellular matrix to meet homoeostatic conditions?^71,72^ Addressing the conserved features and differences in tumour biology can lead to novel research, therapeutics and discoveries that one day could be translated into human and non-human clinical care^61,73^.

## Methods

### Veterinary pathological samples

This research complies with all relevant ethical regulations. Archival samples were obtained from the Zoological Society of London. Tasmanian devil facial tumour disease 1 and 2 (DFT1 and DFT2) and canine transmissible venereal tumour’s samples collection procedures were approved by the University of Cambridge Department of Veterinary Medicine Ethics and Welfare Committee (CR191). All the canine melanoma samples were prepared at the University of Turin, they belong to dogs that were privately owned and sampled for diagnostic purposes. A written informed consent of the owners is always signed; thus, a formal approval of the Institution Committee for Animal Care is not required. All the canine prostate carcinoma were prepared at the University of Queensland, the protocol was approved by Animal Ethics Committee (approval no. ANFRA/SVS/406/13).

In total, 99 H&E samples from 29 species were retrieved from the Zoological Society of London’s (ZSL) pathological archive, derived from clinical or postmortem examinations of ZSL London Zoo’s living collections (Supplementary Table 6). Of these, 51 slides from 22 species passed quality control for image analysis, and 18 slides representing 18 species were selected by the pathologists for subsequent analyses. Exclusion criteria defined for quality control include the presence of haemorrhage, ample necrotic tissue, the lack of tumour components, and the presence of high amounts of melanin/pigments in the tissue samples hindering the correct identification of individual cells. Samples were either obtained through tissue biopsies from surgery or routine postmortem examinations from animals that were (i) examined immediately after euthanasia or (ii) stored at 4 degrees Celsius and examined within two days from death. Lesions were excised, fixed in 10% neutral buffered formalin solution and trimmed before being sent to external institutions (IZVG Pathology and Finn Pathologists) for histopathological processing, paraffin embedding, sectioning, and staining with H&E for analysis. Additionally, four tumour samples from individuals with Tasmanian devil facial tumour disease 1 and 2 (DFT1 and DFT2) and seven with canine transmissible venereal tumour (CTVT) were collected and digitalised from the Transmissible Cancers Group, University of Cambridge. A sample to represent each species was then selected for our analysis; these were previously reported in the following studies: *C. familiaris*^74^ and *S. harrisii*^10^. We refer to this 20 samples cohort as ‘cohort 0’, where each tumour sample is coded according to the scientific name of the species from where it was reported, but not implying the extrapolation to other tumour types for the same species. All slides were scanned using NanoZoomer S210 digital slide scanner (C13239-01) and NanoZoomer digital pathology system v.3.1.7 (Hamamatsu) at 40X magnification (228 nm/pixel resolution).

Two clinical cohorts include survival data associated with tumour tissue sections. A set of 12 H&E slides from 12 dogs with canine prostate tumours (median age 10.25 years) with a median survival time of 108 days was provided by the University of Queensland, Australia. An additional canine melanoma cohort was facilitated by the Department of Veterinary Sciences, University of Torino, Italy, consisting of 88 H&Es from 88 dogs having a median age of 11 years at diagnosis and a median survival time of 370 days. Both canine prostate and melanoma cohorts were utilised for the study of immune response and cancer outcome to provide a more robust analysis with emphasis on a single species and prognostic value of classifying and georeferencing single cells in the tissue. All canine patients were euthanised due to poor clinical conditions or had a tumour-related death; hence, survival data corresponds to overall survival from the moment of tumour diagnosis. All slides were scanned at 40X in the corresponding institutions.

### AI human-lung model

The entire deep-learning-based single-cell analysis pipeline described in^30^ was implemented. This pipeline was designed and developed for human lung tumour specimens. Briefly, all 20 whole-section images were first down-scaled to 20X and then tiled into 2000 × 2000 images for subsequent three-stage analysis. Firstly, all viable H&E tumour tissue areas are segmented. Secondly, within the segmented tissue image, a spatially constrained convolutional neural network predicts for each pixel the probability that it belongs to the centre of a nucleus; cell nuclei were then detected from the probability map obtained from the deep network. Lastly, each identified cell was classified using a neighbouring ensemble predictor coupled with a spatially constrained convolutional neural network. There were four cell classes: cancer (malignant epithelial) cells, lymphocytes (including plasma cells), noninflammatory stromal cells (fibroblasts and endothelial cells) and an ‘other’ cell type that included nonidentifiable cells, less abundant cells such as macrophages and chondrocytes and ‘normal’ pneumocytes and bronchial epithelial cells.

We focused on the three main classes, cancer cells, lymphocytes, and stromal cells. Two board-certified specialist veterinary pathologists (C.P. and K.H.) annotated 14,570 cancer, lymphocyte and stromal cells on raw whole-section images from the pan-species cohort. In the canine prostate cohort, 26,997 annotations were made by C.P. (13,177 cancer cells, 10,312 lymphocytes, and 3508 stromal cells).

The algorithm’s performance for detecting and classifying cells across all species was evaluated directly against the ground truth provided by pathologists’ annotations. Individual class accuracy statistics were calculated using the R function ‘confusionMatrix’ from the R package caret. To analyse the variability in the classification balanced accuracy values, BCAcc, across tumour or cell types, we used a Kruskal–Wallis test. Confidence interval (95%) for effect size (*η*^2^) was estimated via bootstrap. When the test was significant (*p* < 0.05), we applied multiple comparisons correcting *p* values using Bonferroni–Hochberg procedures and estimating the 95% confidence interval for the difference then evaluating if the interval includes 0 under the null hypothesis of no difference (R package emmeans).

Features extraction at the cell level was done with two steps: a MicroNet model^75^ pre-trained on lung H&Es to segment all cells, followed by automatic extraction of morphological measurements for the set of properties from each cell’s mask. This allowed the extraction of 27 features for immune and cancer cells annotated by pathologists in the human and non-human slides (MATLAB function ‘regionprops’ with additional modifications as defined in Supplementary Table 2). Annotated cells were mapped to the segmented cell centroid with a strict threshold of 4 pixels (<2 µm, which is less than 1/3 of a lymphocyte cell) and were visually assessed to confirm correct mapping. Dimension reduction of the 27 cellular features was performed using 2D t-distributed stochastic neighbour embedding (t-SNE, perplexity = 50, theta = 0), enabling us to build a morphological space for each cell class (Fig. 5). We computed morphological space overlap using the R package dynRB, which calculates overlap based on the product of overlap in the two t-SNE dimensions. We calculated the morphospace overlap between animal’s and human’s cancer cells and lymphocytes and compared them using Kruskall–Wallis test followed by multiple comparisons with Benjamini & Hochberg p-value correction.

### Statistical analyses of prognostic value

To test the prognostic value of intratumor immune infiltration in the canine cohorts (prostate tumours and melanomas), we applied multivariate Cox Proportional Hazards considering lymphocytes’ relative abundance, tumour-lymphocytes spatial colocalisation, and age as predictors. The immune estimation metric corresponds to the spatial colocalisation between cancer and immune cells (Morisita-Horn overlap index^35^). Morisita-Horn index goes between 0 and 1; for a better interpretation of the Cox multivariate model, colocalisation was multiplied by 100. We tested in separate analyses the effect of colocalisation as a continuous variable and also dichotomised, considering the lower quartile of the distribution as the cutoff. For visualisation, age-adjusted Kaplan–Meier curves were obtained from the multivariate analyses (R package survminer). To avoid strong multicollinearity between the predictor variables explaining the survival response, we calculated the variance inflation factor (VIF), only keeping variables with VIF < 5. Due to data availability, only for melanoma cases, death related/unrelated to the tumour was considered as a censor variable in the multivariate analysis.

### AI model for canine prostate carcinoma

An alternative model, the canine prostate carcinoma model, was trained to compare the lung model’s performance with a non-human-based model trained with all the single-cell annotations from the prostate cohort and tested on the pan-species cohort. The canine prostate model has the same architecture as the lung model. The evaluation of the performance of this model was done in the same way as the human-lung model on single-cell annotations in the cohort 0.

### Guidelines

Guidelines for slide digitalisation, slide quality control, running the AI model, collecting annotations for validation, and computation of the morphospace overlap index, is available and will be updated through our network webpage www.panspecies.ai/project1atlas.

### Reporting summary

Further information on research design is available in the Nature Portfolio Reporting Summary linked to this article.

## Supplementary information

Supplementary materials

Reporting Summary

## Data Availability

The pan-species digital pathology atlas is publicly available at http://synapse.org/panspecies_ai, providing pan-species digital slide images and pathological annotations of 41,756 single-cell annotations across 20 species. Access can be obtained by registering at http://synapse.org and accepting the Synapse Governance policies for responsible research and data handling. Additionally, slide digitalisation and quality control protocols described in Methods are available at https://www.panspecies.ai/project1atlas. The data used to create figure panels generated in this study are provided in the Supplementary Information/Source Data files. Source data are provided with this paper.

## Source data

Source Data

## Code availability

All code used for statistical analyses of image data and morphospace overlap test tool was developed in R (v.4.0.3), and it is available at https://github.com/simonpcastillo/PanSpeciesHistology^76^. The deep-learning pipeline for digital pathology image analysis is previously available for non-commercial research purposes at https://github.com/qalid7/compath.
