# Supplementary material for: Bridging clinic and wildlife care with AI-powered pan-species computational pathology
Source: Nat Commun. 2023 Apr 26;14:2408. doi: 10.1038/s41467-023-37879-x (PMC10133243; doi:10.1038/s41467-023-37879-x)
Supplement: Supplementary file 1 — Supplementary materials [file 41467_2023_37879_MOESM1_ESM.pdf]

Supplementary materials

Bridging clinic and wildlife care with AI-powered pan-species computational pathology

Authors

Khalid AbdulJabbar[1,2+], Simon P. Castillo[1,2+], Katherine Hughes[3], Hannah Davidson[4,5], Amy M. Boddy[6], Lisa M. Abegglen[7,8], Lucia Minoli[9], Selina Iussich[9], Elizabeth P. Murchison[3], Trevor A. Graham[1,5], Simon Spiro[4], Carlo C. Maley[10], Luca Aresu[9], Chiara Palmieri[11], Yinyin Yuan[1,2*^]

Affiliations

[1]Centre for Evolution and Cancer, The Institute of Cancer Research, London, UK.
[2]Division of Molecular Pathology, The Institute of Cancer Research, London, UK.

[3]Department of Veterinary Medicine, University of Cambridge, Madingley Road, Cambridge, UK.

[4]Zoological Society of London, London, UK.

[5]Centre for Genomics and Computational Biology, Barts Cancer Institute, Queen Mary University of London, Charterhouse Sq, London, UK.

[6]Department of Anthropology, University of California Santa Barbara, Santa Barbara, CA, USA.

[7]Department of Pediatrics and Huntsman Cancer Institute, University of Utah, Salt Lake City, UT, USA.

[8]PEEL Therapeutics, Inc., Salt Lake City, UT, USA.

[9]Department of Veterinary Sciences, University of Turin, 10095 Grugliasco, Italy.

[10] Arizona Cancer Evolution Center, Biodesign Institute and School of Life Sciences, Arizona State University, Tempe, USA.

[11] School of Veterinary Science, The University of Queensland, 4343 Gatton, Queensland, Australia.

[+]Authors contributed equally.

*Correspondence to Yinyin Yuan (yyuan6@mdanderson.org)

^Current affiliation: Department of Translational Molecular Pathology, The University of Texas MD Anderson Cancer Center

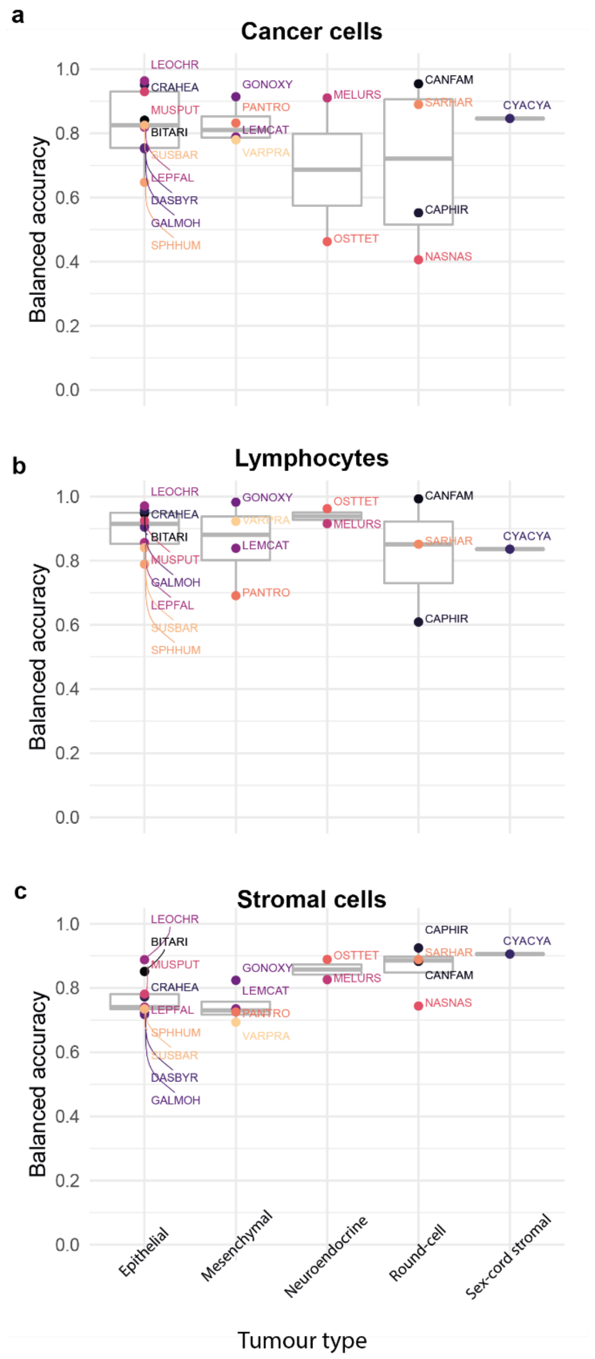

Supplementary Figure 1 Extended AI single-cell prediction comparison across tumour types. Balanced accuracy is computed as the average of sensitivity and specificity for (**a**) cancer, (**b**) lymphocytes and (**c**) stromal cells for all species. Species, n = 20 independent samples, are grouped according to their tumour type and are labelled with their codes, for more species information, see Table 1. Colour-code are for independent samples (one per species). In boxplots, thick horizontal lines indicate the median value, outliers are indicated by the extreme points, the first and third quantiles are represented by the box edges and vertical lines indicate minimum and maximum values.

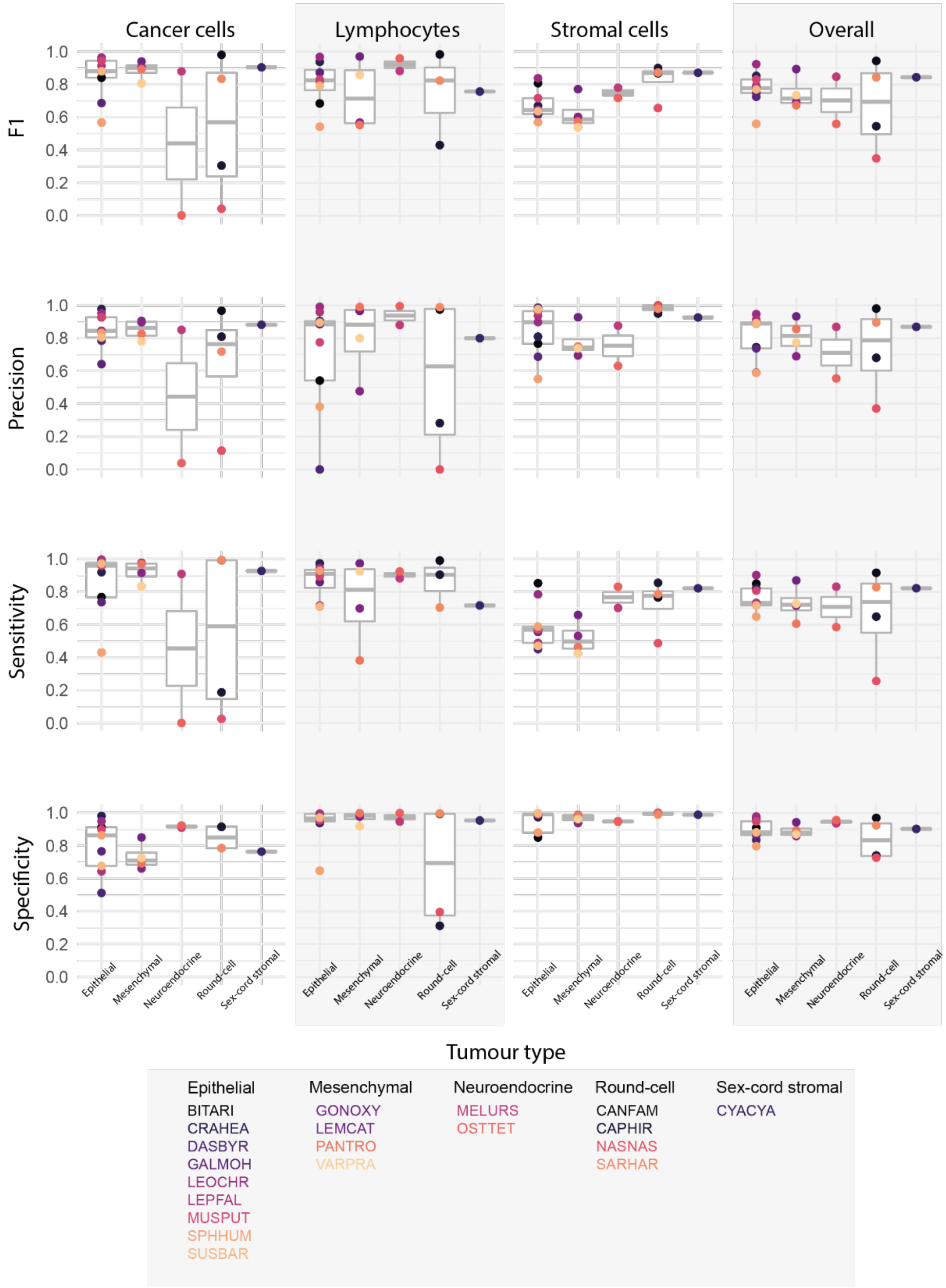

Supplementary Figure 2 Extended AI prediction variability for inter and intra- species tumour microenvironment cells. For each species, four metrics were evaluated including F1, precision, sensitivity, and specificity (rows) for the prediction accuracy of cancer, lymphocytes, and stromal cells as well as their average shown as ‘overall’ (columns). Species are grouped according to their tumour type and are labelled with their codes, for more species information, see Table 1. For the number of cells annotated per cell class for each species, see supplementary table 2. Center line in boxplots indicates median value; box limits are upper and lower quartiles; whiskers indicate minimum and maximum values.

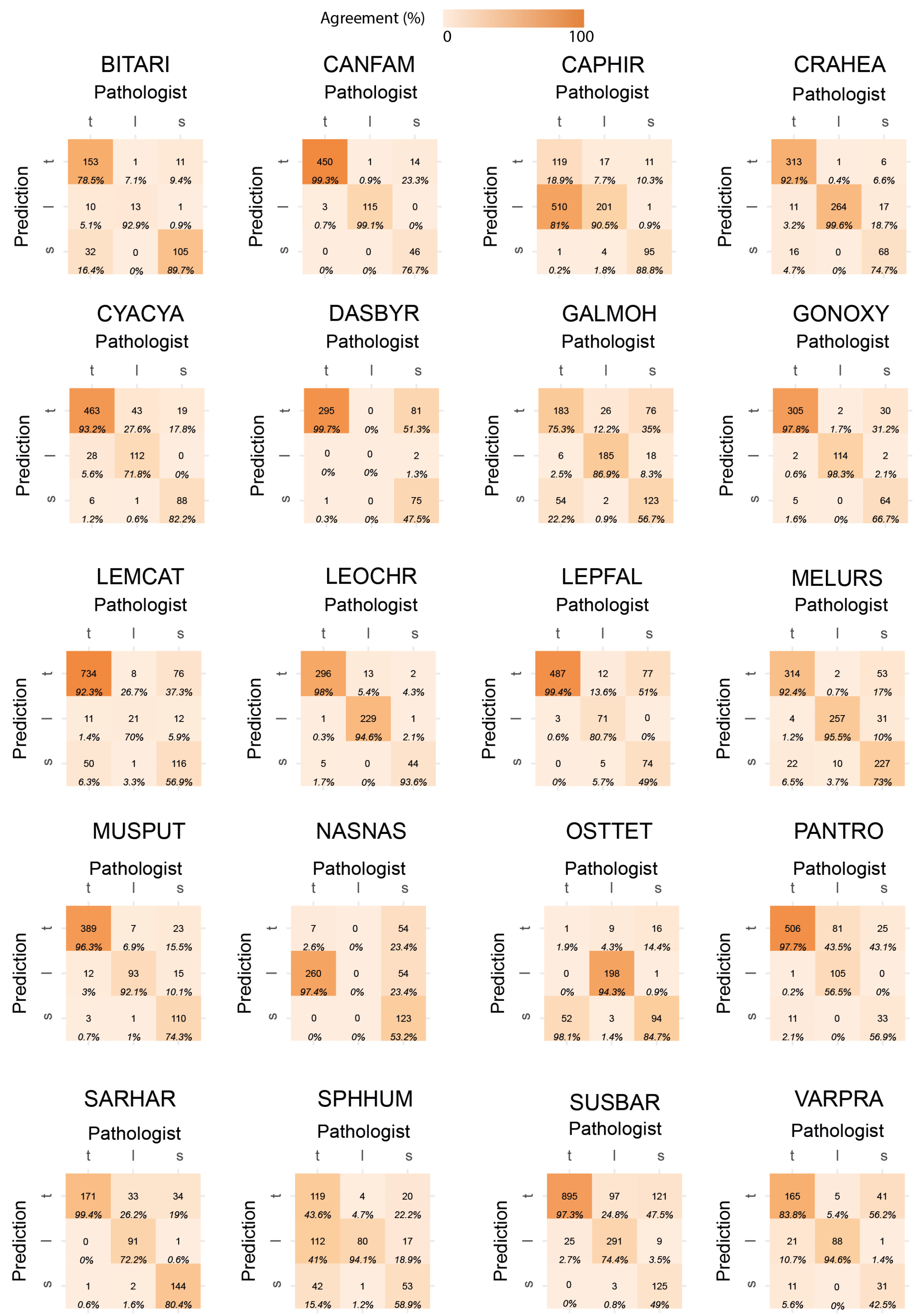

Supplementary Figure 3 Confusion matrices of cell classification. An individual confusion matrix for each sample/species depicts the relationship between pathologist annotations and AI predictions of single-cell classes: lymphocytes (l), stromal cells (s), and tumour cells (t). Each cell in the matrix contains the number of cells for each intersection and the percentage of the pathologist's annotations for each class predicted in different classes by the AI. Colour intensity indicates highest agreement between prediction and pathologist annotations.

Supplementary Table 1 Performance of classification. AUCROC values for each cell class corresponding to each tissue/species. Value between brackets (n) indicates the number pathologists' annotations for each cell class/species.

Species code	Scientific name	Diagnosis	Lymphocytes	Tumour cells	Stromal cells	Mean
BITARI	*Bitis arietans*	Carcinoma	0.947 (14)	0.847 (199)	0.872 (123)	0.888
CANFAM	*Canis familiaris*	CTVT	0.993 (116)	0.954 (453)	0.883 (60)	0.943
CAPHIR	*Capra hircus*	Lymphoma	0.606 (222)	0.552 (632)	0.941 (111)	0.700
CRAHEA	*Crateromys heaneyi*	Hepatocellular carcinoma	0.966 (271)	0.95 (340)	0.86 (119)	0.925
CYACYA	*Cyanerpes cyaneus*	Sertoli cell tumor	0.836 (156)	0.848 (499)	0.906 (107)	0.863
DASBYR	*Dasyuroides byrnie*	Squamous cell carcinoma	-	0.742 (296)	0.736 (166)	0.739
GALMOH	*Galago moholi*	Squamous cell carcinoma	0.908 (215)	0.758 (248)	0.722 (221)	0.796
GONOXY	*Gonyosoma oxycephala*	Metastatic anaplastic sarcoma	0.986 (117)	0.913 (312)	0.827 (97)	0.909
LEMCAT	*Lemur catta*	Haemangiosarcom a	0.838 (30)	0.782 (801)	0.753 (218)	0.791
LEOCHR	*Leontopithecus chrysomelas*	Adenoma	0.97 (242)	0.959 (303)	0.964 (56)	0.964
LEPFAL	*Leptodactylus fallax*	Adenocarcinoma	0.901 (99)	0.811 (490)	0.741 (151)	0.818
MELURS	*Melursus ursinus inornatus*	Pheochromocytom a	0.949 (291)	0.915 (345)	0.839 (323)	0.901
MUSPUT	*Mustela putorius furo*	Sebaceous epithelioma	0.936 (104)	0.921 (405)	0.868 (193)	0.908
NASNAS	*Nasua nasua*	Lymphoma	-	0.396 (268)	0.766 (252)	0.581
OSTTET	*Osteolaemus t. tetraspis*	Lipoma	0.968 (214)	0.47 (815)	0.819 (113)	0.753
PANTRO	*Pan troglodytes*	Spindle cell tumor	0.781 (274)	0.771 (521)	0.777 (71)	0.776
SARHAR	*Sarcophilus harrisii*	Devil facial tumor 1 (DFT1)	0.86 (129)	0.887 (172)	0.897 (183)	0.881
SPHHUM	*Spheniscus*	Renal cell adenoma	0.793 (86)	0.649 (276)	0.734 (90)	0.726

	humboldti					
SUSBAR	*Sus barbatus*	Adenocarcinoma	0.858 (410)	0.818 (920)	0.744 (265)	0.806
VARPRA	*Varanus prasinus*	Spindle cell sarcoma	0.932 (95)	0.78 (198)	0.693 (73)	0.802
		mean	0.891	0.786	0.817	

Supplementary Table 2 Performance of AI human-lung model on canine prostate carcinoma cohort based on 26,997 pathologist annotations.

Overall performance			
Accuracy (95% CI)	0.877 (0.873, 0.881)		
Mean balanced accuracy (BCAcc)	0.9		
Kappa	0.7978		
Class-specific performance			
	Tumour cells	Lymphocytes	Stromal cells
Sensitivity	0.866	0.911	0.815
Specificity	0.899	0.982	0.930
Balanced Accuracy	0.883	0.947	0.872
Precision	0.892	0.969	0.635
F1	0.879	0.939	0.714
AUROC	0.875	0.936	0.872

Supplementary Table 3 Performance comparison between the AI human-lung and canine-prostate models in predicting cell classes of cohort 0. Values correspond to the average balanced accuracy across the three classes (Lymphocytes, Tumour cells, Stromal cells)

					Balanced Accuracy	
Species code	Scientific name	Diagnosis	Neoplasia site	Tumour type	Lung model	Dog model
BITARI	*Bitis arietans*	Carcinoma	Pancreas	Epithelial	0.88	0.535
CANFAM	*Canis familiaris*	Canine transmissible venereal tumor	Intra vaginal	Round-cell	0.94	0.533
CAPHIR	*Capra hircus*	Lymphoma	Forestomach	Round-cell	0.70	0.534
CRAHEA	*Crateromys heaneyi*	Hepatocellular carcinoma	Liver	Epithelial	0.89	0.714
CYACYA	*Cyanerpes cyaneus*	Sertoli cell tumor	Testis	Sex-cord stromal	0.86	0.496
DASBYR	*Dasyuroides byrnie*	Squamous cell carcinoma	Mouth	Epithelial	0.74	0.617
GALMOH	*Galago moholi*	Squamous cell carcinoma	Skin	Epithelial	0.79	0.728
GONOXY	*Gonyosoma oxycephala*	Metastatic anaplastic sarcoma	Multiple	Mesenchymal	0.91	0.586
LEMCAT	*Lemur catta*	Haemangiosarcoma	Kidney	Mesenchymal	0.79	0.728
LEOCHR	*Leontopithecus chrysomelas*	Adenoma	Pituitary	Epithelial	0.94	0.68
LEPFAL	*Leptodactylus fallax*	Adenocarcinoma	Celomic cavity	Epithelial	0.81	0.587
MELURS	*Melursus u. inornatus*	Pheochromocytoma	Adrenal	Neuroendocrine	0.88	0.581
MUSPUT	*Mustela putorius furo*	Sebaceous epithelioma	Skin	Epithelial	0.88	0.698
NASNAS	*Nasua nasua*	Lymphoma	Multiple	Round-cell	0.57	0.461
OSTTET	*Osteolaemus t. tetraspis*	Lipoma	Liver	Neuroendocrine	0.77	0.797
PANTRO	*Pan troglodytes*	Spindle cell tumor	Palate	Mesenchymal	0.75	0.738
SARHAR	*Sarcophilus harrisii*	Devil facial tumor 1 (DFT1)	Hard palate near left side	Round-cell	0.88	0.559
SPHHUM	*Spheniscus humboldti*	Renal cell adenoma	Kidney	Epithelial	0.72	0.503
SUSBAR	*Sus barbatus*	Adenocarcinoma	Uterus	Epithelial	0.80	0.681

VARPRA	*Varanus prasinus*	Spindle cell sarcoma	Multiple	Mesenchymal	0.80	0.552

Supplementary Table 4 The 27 single-cell features extracted to compute the morphological space.

Feature	Description
Area	Two-dimensional extension of a shape
MajorAxisLength	Longest diameter
MinorAxisLength	Shortest diameter
Eccentricity	Magnitude inversely related to shape curvature
ConvexArea	Area resulting from connecting the external points of the shape
FilledArea	Area of a corresponding image with holes filled in
EquivDiameter	Diameter of a circle with the same area as the region
Solidity	Extent to which the shape fills the convex area
Extent	Ratio of pixels in the region to pixels in the total bounding box
Perimeter	Length of the shape boundary
ConvexHullMean	Smallest convex polygon that can contain the region
FilledImageMean	Average of pixels corresponding to the segmented mask, with all holes filled
ConvexImageMean	Average of pixels corresponding to a segmented mask which specifies the convex hull of the region
Diameters	Cell diameter using major and minor axes
Radii	Cell radius
MeanIntensity_R	Mean pixel intensity in the red channel
MinIntensity_R	Minimum pixel intensity in the red channel
MaxIntensity_R	Maximum pixel intensity in the red channel
MeanIntensity_G	Mean pixel intensity in the green channel
MinIntensity_G	Minimum pixel intensity in the green channel

MaxIntensity_G	Maximum pixel intensity in the green channel
MeanIntensity_B	Mean pixel intensity in the blue channel
MinIntensity_B	Minimum pixel intensity in the blue channel
MaxIntensity_B	Maximum pixel intensity in the blue channel
RGBMeanIntensity	Mean pixel intensity in the composed image
RGBMinIntensity	Minimum pixel intensity in the composed image
RGBMaxIntensity	Maximum pixel intensity in the composed image

Supplementary Table 5 Morphological overlap for each species of the cohort 0 with other cell classes. Each column has the ratio of the morphospace for the focal cell class overlapped with another cell class. Prefix *a-* and *h-* means non-human and human cell classes, respectively. *tum* refers to tumour cells and *lymp* to lymphocytes.

Species code	a- tumour/ h- tumour	a-tumour/ h-lymphocyte	a- tumour / a-lymphocyte	a-lymphocyte/ h- tumour	a-lymphocyte/ h-lymp	a-lymphocyte/ a- tumour
BITARI	0.703	0.362	0.101	0.291	0.883	0.340
CANFAM	0.807	0.108	0.013	0.189	0.933	0.034
CAPHIR	0.243	0.848	0.355	0.171	0.851	0.938
CRAHEA	0.514	0.046	0.004	0.200	0.925	0.006
CYACYA	0.668	0.384	0.247	0.223	0.819	0.233
DASBYR	0.513	0.068	-	-	-	-
GALMOH	0.431	0.217	0.069	0.172	0.780	0.282
GONOXY	0.914	0.244	0.170	0.212	0.718	0.243
LEMCAT	0.691	0.094	0.017	0.306	0.911	0.052
LEOCHR	0.737	0.228	0.031	0.128	0.538	0.163
LEPFAL	0.561	0.306	0.039	0.234	0.913	0.624
MELURS	0.491	0.132	0.020	0.166	0.854	0.042
MUSPUT	0.413	0.461	0.098	0.156	0.782	0.434
NASNAS	0.144	0.740	-	-	-	-
OSTTET	0.487	0.443	0.082	0.175	0.844	0.464
PANTRO	0.483	0.056	0.070	0.775	0.252	0.153

SARHAR	0.700	0.064	0.026	0.357	0.839	0.034
SPHHUM	0.439	0.651	0.169	0.149	0.481	0.122
SUSBAR	0.559	0.139	0.089	0.267	0.867	0.156
VARPRA	0.797	0.272	0.049	0.161	0.729	0.103

Supplementary Table 6 Summary of sample preparation methods as provided from the Zoological Society of London's pathological archive.

Case ID	Species code	Pathologists	Method
B01/17	MUSPUT	IZVG/RVC- DD	Biopsy: removed during surgery and formalin-fixed
B02/18	GALMOH	IZVG/RVC- DD	Biopsy: removed during surgery and formalin-fixed
B04/17	LEMCAT	IZVG/RVC- MS	Biopsy: removed during surgery and formalin-fixed
B07-8/04	PANTRO	ZSL- AP	Biopsy - removed during surgery and formalin-fixed
B09/04	DASBYR	ZSL- AP	Biopsy - removed during surgery and formalin-fixed
W17M035	MELURS	IZVG/RVC- MS	Euthanasia: Carcass fresh – PM examination one day after death
W17R187	OSTTET	IZVG/RVC- DD	Natural death: Carcass fresh - PM on day of death
ZA1360/15	LEPFAL	IZVG/RVC- MS	Natural death: Carcass slightly autolysed – PM on day of death
ZB017/18	CYACYA	IZVG/RVC- MS	Euthanasia: Carcass fresh – PM examination one day after death
ZB485/19	SPHHUM	IZVG/RVC- CS	Euthanasia: No comment on carcass condition - PM carried out 2 days after euthanasia
ZM134/17	CAPHIR	IZVG/RVC- CS	Carcass fresh – euthanised and PM'd on day of death
ZM138/17	SUSBAR	IZVG/RVC- MS	Carcass fresh - PM on day of death
ZM203/17	LEOCHR	IZVG/RVC- MS	Carcass fresh - PM on day of death
ZM633/18	CRAHEA	IZVG/RVC- DD	Euthanasia: Carcass fresh – PM examination one day after death
ZM748/18	NASNAS	IZVG/RVC- CS	Euthanasia: Carcass fresh – PM examination one day after death
ZR1145/15	GONOXY	IZVG/RVC- MS	Euthanasia: Carcass fresh - kept in fridge two days before examination
ZR1148/18	VARPRA	IZVG/RVC- DD	Euthanasia: Carcass fresh – PM examination one day after death
ZR474/19	BITARI	IZVG/RVC- CS	Euthanasia: Carcass fresh – PM examination one day after death
