# Supplementary material for: Bridging clinic and wildlife care with AI-powered pan-species computational pathology
Source: Nat Commun. 2023 Apr 26;14:2408. doi: 10.1038/s41467-023-37879-x (PMC10133243; doi:10.1038/s41467-023-37879-x)
Supplement: Supplementary file 2 — Reporting Summary [file 41467_2023_37879_MOESM2_ESM.pdf]

nature portfolio

Corresponding author(s): Yinyin Yuan

Last updated by author(s): Jan 13, 2023

Reporting Summary

Nature Portfolio wishes to improve the reproducibility of the work that we publish. This form provides structure for consistency and transparency in reporting. For further information on Nature Portfolio policies, see our Editorial Policies and the Editorial Policy Checklist.

Statistics

For all statistical analyses, confirm that the following items are present in the figure legend, table legend, main text, or Methods section.

n/a	Confirmed	
☐	☒	The exact sample size (n) for each experimental group/condition, given as a discrete number and unit of measurement
☐	☒	A statement on whether measurements were taken from distinct samples or whether the same sample was measured repeatedly
☐	☒	The statistical test(s) used AND whether they are one- or two-sided<br>*Only common tests should be described solely by name; describe more complex techniques in the Methods section.*
☐	☒	A description of all covariates tested
☐	☒	A description of any assumptions or corrections, such as tests of normality and adjustment for multiple comparisons
☐	☒	A full description of the statistical parameters including central tendency (e.g. means) or other basic estimates (e.g. regression coefficient) AND variation (e.g. standard deviation) or associated estimates of uncertainty (e.g. confidence intervals)
☐	☒	For null hypothesis testing, the test statistic (e.g. F, t, r) with confidence intervals, effect sizes, degrees of freedom and P value noted<br>*Give P values as exact values whenever suitable.*
☒	☐	For Bayesian analysis, information on the choice of priors and Markov chain Monte Carlo settings
☒	☐	For hierarchical and complex designs, identification of the appropriate level for tests and full reporting of outcomes
☐	☒	Estimates of effect sizes (e.g. Cohen's d, Pearson's r), indicating how they were calculated

Our web collection on statistics for biologists contains articles on many of the points above.

Software and code

Policy information about availability of computer code

Data collection	All slides were scanned using NanoZoomer S210 digital slide scanner (C13239-01) and NanoZoomer digital pathology system v.3.1.7 (Hamamatsu) at 40X (228nm/pixel resolution). The entire deep learning-based single-cell analysis pipeline described in AbdulJabbar, K. et al. (Nat Medicine 26, 1054–1062, 2020) was implemented.
Data analysis	The deep-learning pipeline for digital pathology image analysis is previously available for non-commercial research purposes at https://github.com/qalid7/compath. All code used for statistical analyses of image data and morphospace overlap test tool was developed in R (v.4.0.3) and it is available at https://github.com/simonpcastillo/PanSpeciesHistology.

For manuscripts utilizing custom algorithms or software that are central to the research but not yet described in published literature, software must be made available to editors and reviewers. We strongly encourage code deposition in a community repository (e.g. GitHub). See the Nature Portfolio guidelines for submitting code & software for further information.

Data

Policy information about availability of data

All manuscripts must include a data availability statement. This statement should provide the following information, where applicable:

- Accession codes, unique identifiers, or web links for publicly available datasets
- A description of any restrictions on data availability
- For clinical datasets or third party data, please ensure that the statement adheres to our policy

The pan-species digital pathology atlas is publicly available for registered users of Synapse at http://synapse.org/panspecies_ai, providing pan-species digital slide images and pathological annotations of 41,756 single-cell annotations across 20 species. The pan-species digital pathology atlas data are publicly available, access

can be obtained by registering at http://synapse.org accepting the Synapse Governance policies for responsible research and data handling. Additionally, slide digitalisation and quality control protocols complementary to the present in Methods are available at https://www.panspecies.ai/project1atlas and will be updated regularly. Source data are provided with this paper. The animal data generated in this study are provided as a Source Data file.

Field-specific reporting

Please select the one below that is the best fit for your research. If you are not sure, read the appropriate sections before making your selection.

- [x] Life sciences
- [ ] Behavioural & social sciences
- [ ] Ecological, evolutionary & environmental sciences

For a reference copy of the document with all sections, see nature.com/documents/nr-reporting-summary-flat.pdf

Life sciences study design

All studies must disclose on these points even when the disclosure is negative.

Sample size

In total, 99 H&E samples from 29 species were identified from the Zoological Society of London's (ZSL) pathological archive, derived from clinical or postmortem examinations of ZSL London Zoo's living collections (Table S1). Of these, 51 slides from 22 species passed quality control for image analysis, and 18 slides representing 18 species were selected by the pathologists for subsequent analyses. In addition, two samples were provided by the Transmissible Cancer Group, University of Cambridge, as previously reported. In adition, a set of 12 H&E slides from 12 dogs with canine prostate tumours (median age 10.25 years) with a median survival time of 108 days was provided by the University of Queensland, Australia. The melanoma cohort was facilitated by the Department of Veterinary Sciences, University of Torino, Italy, consisting of 88 H&Es from 88 dogs having a median age of 11 years at diagnosis and a median survival time of 370 days. Both canine prostate and melanoma cohorts were utilized for the study of immune response and cancer outcome to provide a more robust analysis with emphasis on a single species and prognostic value of classifying and georeferencing single cells in the tissue. All canine patients were euthanised due to poor clinical conditions or had a tumour-related death; hence, survival data corresponds to overall survival from the moment of tumour diagnosis. All slides were scanned at 40x in the corresponding institutions.

Prospective sample size calculation was not computed. Considering the limited samples available in veterinary pathology, the sample size consisted in all the available samples of each cohort that passed quality control as described in methods.

Data exclusions

Exclusion criteria were the lack of tumour components and the presence of high amounts of melanin/pigments in the tissue samples hindering the correct identification of individual cells.

Replication

For reproducibility the codes are openly available. The AI model has been previously validated and published. In addition, upon publication, we will provide the corresponding digital slide images and pathological annotations of 14,570 single-cell annotations across 20 species.

Randomization

This study does not include experimental manipulation, hence no experimental groups, were defined.

Blinding

Pathologist were blind to the model prediction, they did not have influence on the development of the image analysis pipeline or the statistical tests to evaluate model's performance.

Reporting for specific materials, systems and methods

We require information from authors about some types of materials, experimental systems and methods used in many studies. Here, indicate whether each material, system or method listed is relevant to your study. If you are not sure if a list item applies to your research, read the appropriate section before selecting a response.

Materials & experimental systems

n/a	Involved in the study	
☒	☐	Antibodies
☒	☐	Eukaryotic cell lines
☒	☐	Palaeontology and archaeology
☐	☒	Animals and other organisms
☒	☐	Human research participants
☒	☐	Clinical data
☒	☐	Dual use research of concern

Methods

n/a	Involved in the study	
☒	☐	ChIP-seq
☒	☐	Flow cytometry
☒	☐	MRI-based neuroimaging

Animals and other organisms

Policy information about studies involving animals; ARRIVE guidelines recommended for reporting animal research

Laboratory animals

This study did not involve laboratory animals

Wild animals

This study did not involve direct manipulation of wild animals

Field-collected samples	We analysed digital images from archival records from the the Zoological Society of London's pathological archive and provided by the Transmissible Cancer Group (University of Cambridge), University of Turin, and Queensland University.
Ethics oversight	Our analysis was made on postmortem samples managed by Zoological Society of London's pathological archive and by the Transmissible Cancer Group, University of Cambridge, University of Turin, and Queensland University. This research complies with all relevant ethical regulations. Archival samples were obtained from the Zoological Society of London. Tasmanian devil facial tumour disease 1 and 2 (DFT1 and DFT2) and canine transmissible venereal tumour's samples collection procedures were approved by the University of Cambridge Department of Veterinary Medicine Ethics and Welfare Committee (CR191). All the canine melanoma samples were prepared at the University of Turin, they belong to dogs that were privately owned and sampled for diagnostic purposes. A written informed consent of the owners is always signed; thus, a formal approval of the Institution Committee for Animal Care is not required. All the canine prostate carcinoma were prepared at the University of Queensland, the protocol was approved by Animal Ethics Committee (approval no. ANFRA/SVS/406/13).

Note that full information on the approval of the study protocol must also be provided in the manuscript.
